# Occurrence of Luteolin in the Greek Flora, Isolation of Luteolin and Its Action for the Treatment of Periodontal Diseases

**DOI:** 10.3390/molecules28237720

**Published:** 2023-11-22

**Authors:** Athanasios S. Arampatzis, Aspasia Pampori, Eleftheria Droutsa, Maria Laskari, Panagiotis Karakostas, Lazaros Tsalikis, Panagiotis Barmpalexis, Christos Dordas, Andreana N. Assimopoulou

**Affiliations:** 1 School of Chemical Engineering, Aristotle University of Thessaloniki, 54124 Thessaloniki, Greece; arampatzisa@cheng.auth.gr (A.S.A.); aspapamp@gmail.com (A.P.); eleftheriadroutsa@gmail.com (E.D.); 2 Natural Products Research Center of Excellence (NatPro-AUTH), Center for Interdisciplinary Research and Innovation, Aristotle University of Thessaloniki, 57001 Thessaloniki, Greece; pbarmp@pharm.auth.gr; 3 School of Agriculture, Aristotle University of Thessaloniki, 54124 Thessaloniki, Greece; marialaskari00@gmail.com (M.L.); chdordas@agro.auth.gr (C.D.); 4 School of Dentistry, Aristotle University of Thessaloniki, 54124 Thessaloniki, Greece; karako.dent@gmail.com (P.K.); tsalikis@dent.auth.gr (L.T.); 5 Laboratory of Pharmaceutical Technology, Division of Pharmaceutical Technology, School of Pharmacy, Faculty of Health Sciences, Aristotle University of Thessaloniki, 54124 Thessaloniki, Greece

**Keywords:** extraction, aromatic, medicinal plants, secondary metabolites, pharmaceutical use

## Abstract

Higher plants possess the ability to synthesize a great number of compounds with many different functions, known as secondary metabolites. Polyphenols, a class of flavonoids, are secondary metabolites that play a crucial role in plant adaptation to both biotic and abiotic environments, including UV radiation, high light intensity, low/high temperatures, and attacks from pathogens, among others. One of the compounds that has received great attention over the last few years is luteolin. The objective of the current paper is to review the extraction and detection methods of luteolin in plants of the Greek flora, as well as their luteolin content. Furthermore, plant species, crop management and environmental factors can affect luteolin content and/or its derivatives. Luteolin exhibits various biological activities, such as cytotoxic, anti-inflammatory, antioxidant and antibacterial ones. As a result, luteolin has been employed as a bioactive molecule in numerous applications within the food industry and the biomedical field. Among the different available options for managing periodontitis, dental care products containing herbal compounds have been in the spotlight owing to the beneficial pharmacological properties of the bioactive ingredients. In this context, luteolin’s anti-inflammatory activity has been harnessed to combat periodontal disease and promote the restoration of damaged bone tissue.

## 1. Introduction

Higher plants produce, through the photosynthesis process, all the necessary substances for their growth and also for all the other life forms of nature. These are called ‘primary metabolites’. In addition, plants have the ability to biosynthesize a large number of substances that have specific functions and are known as secondary metabolites. Polyphenols are one of the most common groups of secondary metabolites widely distributed in all plant species [1]. The content of polyphenols increases in response to various factors, such as ultraviolet (UV) radiation, high light intensity, low/high temperature, salinity, drought, etc. These conditions cause the creation of free oxygen and nitrogen radicals due to the stress they cause to the plants. One of the functions of polyphenols is the reduction of the effect caused by the presence of free radicals [2]. A class of polyphenols are flavonoids, which are the largest group of phenolic compounds, accounting for more than 5000 different compounds present in plant species [3]. In the last decade, flavonoids have been studied systematically due to experimental and clinical research as they are used as anti-cancer, antioxidant, anti-inflammatory, and antiviral compounds. They also act as cardioprotective, neuroprotective and chemoprotective agents [4].

**Figure 1:**
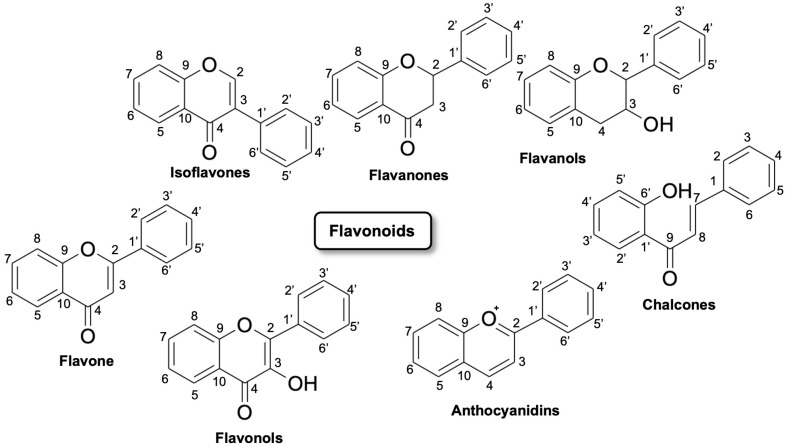
Flavonoids classification and their possible chemical structure [7].

Flavonoids are polyhydroxy–phenolic compounds produced through the phenylpropanoid biosynthetic pathway in plants [5]. They have 15 carbon atoms (phenolic compounds of the type C6–C3–C6) with the structure of two benzene rings joined by a heterocyclic oxygen-centered ring. Flavonoids are divided into seven major subclasses: flavan-3-ols, flavones, flavonols, flavanones, anthocyanins, chalcones and isoflavonoids (Figure 1). Flavonoids include, in particular, flavones, such as luteolin and tetramethoxyluteolin. Flavones are structurally characterized by a double bond and an oxygen atom in the heterocyclic ring C of the flavonoid skeleton. Flavones, such as apigenin and luteolin, can be found in plants showing a wide range of substitutions, including methylations, hydroxylations, acylations, and glycosylations leading mainly to O- or C-glycosides [6].

**Figure 2:**
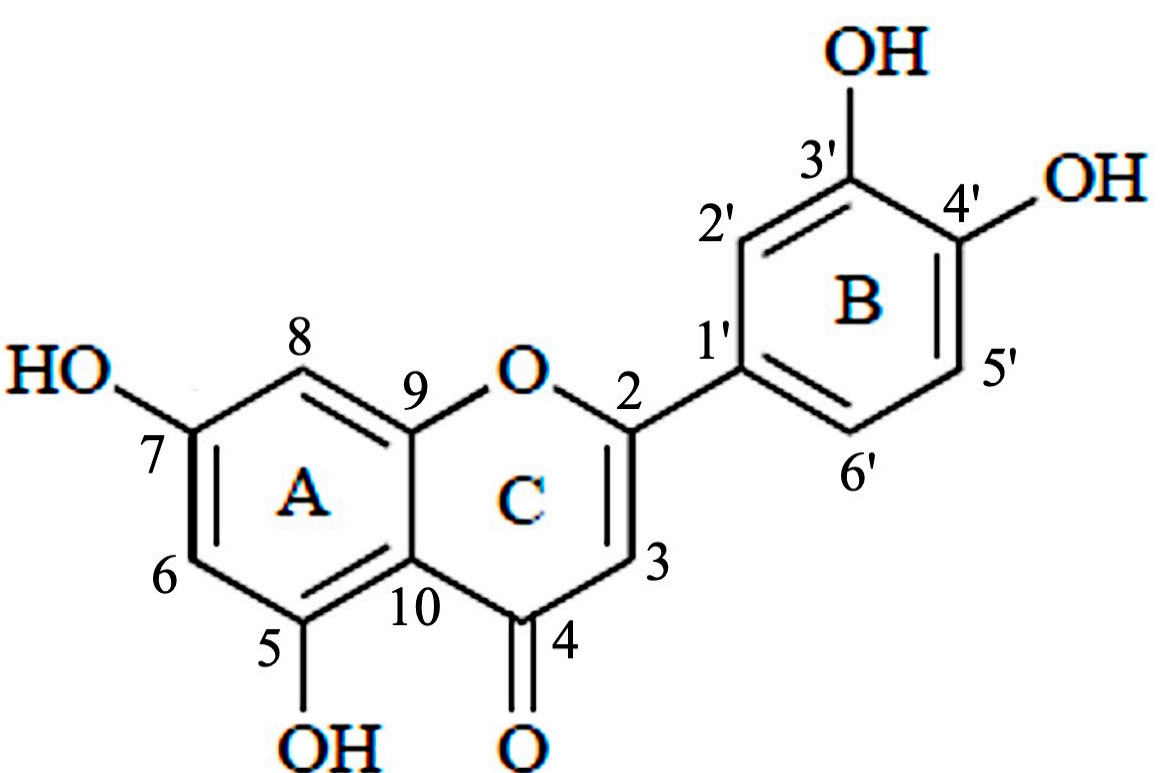
Structure of luteolin.

Luteolin (3′,4′,5,7-tetrahydroxyflavone) belongs to a group of naturally occurring compounds called flavonoids, which are naturally found in several plant species. Because of their abundance in foods, e.g., vegetables, fruits, and medicinal plants, flavonoids are common compounds that act as antioxidants, estrogenic regulators, and antimicrobial agents [8]. Chemically, luteolin has a C6–C3–C6 structure that contains two benzene rings and one oxygen-containing ring with a C2–C3 carbon double bond (Figure 2). Structure-activity relationship studies have shown that the presence of hydroxyl moieties at carbons 5, 7, 3′ and 4′ positions of the luteolin structure and the presence of the 2−3 double bond are responsible for its multiple pharmacological effects [9]. The hydroxyl moieties and the 2–3 double bonds are important structural features in luteolin that are associated with its biochemical and biological activities (anti-cancer, antioxidant, anti-inflammatory, neuroprotective, etc.) [10]. As in other flavonoids, luteolin is often glycosylated in plants, and the glycoside is hydrolyzed to free luteolin during absorption [11]. 

In this review, we focus on the plant species of the Mediterranean area (especially the Greek flora where luteolin has been reported to be present), exploring whether cultivation techniques can affect its content. In addition, the methods for isolating luteolin along with its mechanism of action in the treatment of periodontitis are reviewed.

## 2. Determination of Luteolin Content in Plants of the Greek Flora

Areas that have favorable climate conditions for plant species [12], such as Greece, are ideal for the cultivation of aromatic and medicinal plants [13]. Aromatic and medicinal plants are important for the protection of the environment. In particular, since ancient times, they have been used as fresh, frozen or dry essential oils, originally for the food, pharmaceutical and cosmetic industries [12]. It is estimated that 50,000–70,000 species of higher plants may be used in traditional and modern medicinal systems throughout the world, and about 3000 belong to the group of medicinal and aromatic plants [14]. Aromatic plants contain chemical substances, such as essential oils, polyphenols, glycosides, quinones, flavonols/flavonoids, terpenes, alkaloids, polypeptides or their oxygen-substituted derivatives [15,16]. Some bioactive compounds have therapeutic value, such as antioxidant and antiseptic activities and may reduce the risk of cancer or cardiovascular diseases and treatment of respiratory diseases, stomach or inflammatory disorders [17,18]. Luteolin is a common flavonoid that belongs to the subclass of flavones and exists in many types of plants, including fruits, vegetables, and medicinal herbs [9]. Luteolin, in medicinal plants, is most commonly found in leaves, having been isolated from many plants [19]. The major natural sources of luteolin, based on the literature, are celery, thyme, dandelion, clover flower, chamomile, carrots, peppers, olive oil, peppermint, thyme, rosemary, oregano, and parsley [20,21]. Luteolin is a powerful antioxidant with anti-inflammatory properties that can be used to treat diseases such as periodontitis [22,23]. Table 1 summarizes the major plant species of the Greek flora that contain luteolin and its derivatives. 

The olive tree (*Olea europaea*, Oleaceae), along with its fruit (olive) and leaves, is considered to be very important, exhibiting high nutritional and medicinal value. Besides the main bioactive compound oleuropein, olive drupes and leaves have been found to contain luteolin and its derivatives, among other compounds. Blekas and co-workers (2002) studied different samples of table olives obtained from the retail market that were representative of the main Greek cultivars Conservolea (Amfissa), Nychati (Kalamata) and Chalkidiki. The analyses—using a high-performance liquid chromatography system coupled with diode array detector (HPLC-DAD) and reference compounds—showed that luteolin was among the most abundant phenols in all samples, yet Kalamata olives contained the highest quantity [36]. Luteolin was also detected in olive oils that were extracted from various Greek cultivars Koroneiki, Tsounati, Adramitini, Throubolia, Native from Zakynthos, Lianolia, Asprolia, and Thiaki [35,37,39]. The identification of luteolin was performed either using NMR [35,37] or HPLC-UV and LC-DAD-mass spectrometry (MS) [39]. Moreover, luteolin and luteolin 7-*O*-glucoside were determined in fresh olive fruits, as well as in Greek-style and Spanish-style processed olives from the Kalamon cultivar. Luteolin 7-*O*-glucoside was identified and quantified by means of HPLC-UV [42], as well as HPLC-DAD and LC-(ESI)-MS/MS [41]. In another study, olive leaves from the Greek cultivars Koroneiki, Megaritiki and Kalamon were examined and found to contain luteolin 7-*O*-glucoside. LC-(ESI)-MS/MS method was employed to determine glycoside content in the olive leaves [38]. 4-*O*-glucoside and 7-*O*-glucoside of luteolin were present in the leaves and drupes of samples collected from several major Greek olive varieties, such as Koroneiki, Lianolia Kerkyras, Mastoidis, Adramytini, Megaritiki, Gaidourelia, Kalamata, Konservolia, and Chalkidiki. The two metabolites were identified and quantified by using reference compounds and HPLC-UV analysis [40].

The Lamiaceae family constitutes one of the most significant botanical families, including a large number of aromatic and medicinal plants, such as *Origanum vulgare*, *Rosmarinus officinalis*, *Salvia officinalis* and *Thymus vulgaris*, among others. Luteolin and/or its derivatives have been identified in various plant species of the Lamiaceae family. As mentioned above, luteolin was detected in samples collected from different sites in Greece regarding the following species: *O. vulgare* [32,43], *R. officinalis* [46], *S. officinalis* [46], *S. fruticosa* [43], and *Thymus vulgaris* [46]. The above analyses were performed by means of HPLC-DAD and using reference compounds. Additionally, luteolin was present in *Mentha spicata* [32], *Nepeta cataria* and *N. sibthorpii* [33,51], *Teucrium chamaedrys* [46] and *T. polium* [27]. Other studies have reported that various luteolin derivatives have also been detected in species belonging to the Lamiaceae family. Specifically, 6-hydroxy luteolin 7-*O*-glucoside, luteolin 7-*O*-glucuronide, luteolin 7-*O*-rutinoside, luteolin diglucuronide, 6-methoxyluteolin 7-*O*-glucoside, and 6-methoxyluteolin derivative were tentatively identified via HPLC-DAD and LC-(ESI)-MS/MS methods in *Salvia fruticosa* and *S. pomifera* [48,49]. Choulitoudi et al. reported that *Satureja thymbra* was found to contain luteolin 7,4′-di-*O*-glucuronide, 6-OH luteolin 7,3′-dimethyl ether and 6-OH luteolin 7,3′,4′-trimethyl ether. The above components were identified through HPLC-DAD-ESI-MS/MS analysis [50].

Members of the Asteraceae family were analyzed through NMR and MS methods for their flavonoid content, and it was reported that luteolin was identified in *Crepis incana* [26] and *Cynara* species [29]. Moreover, the derivatives luteolin 7-*O*-rutinoside, luteolin 7-*O*-glucoside, luteolin 7-*O*-malonylglycoside, luteolin 7-*O*-β-d-glucoside, luteolin 7-*O*-β-d-rutinoside, and luteolin 7-*O*-β-gentiobioside, were also present in *Cynara cardunculus*, *Cynara humilis* and *Cynara cornigera* [28,29].

Different research groups have detected luteolin and its derivatives (luteolin 7-*O*-glucoside, 3′-methyl-luteolin) in *Cuminum cyminum* [27], *Petroselinum crispum* [45] and *Petroselinum sativum* [46]. Other sources of luteolin that have been described in the literature are *Asphodelus ramosus* [25], *Juglans regia* [30], *Laurus nobilis* [31], *Pistacia lentiscus* [47], and *Spartium junceum* [27]. Finally, luteolin and its derivatives luteolin 3′-*O*-β-d-glucoside and luteolin 3′,4′-di-*O*-β-d-glucopyranoside were identified by ultra-high-performance LC-high resolution MS (UHPLC-HRMS) analysis in *Paeonia clusii* [44]. 

## 3. Effect of Crop Management on Luteolin Content

Luteolin’s concentration, as well as the quantitative and qualitative composition of the products of secondary metabolites, depends on the genotype of the plant, the crop management (fertilization, irrigation), the growth stage of the plant, soil and climate conditions such as ultraviolet (UV) radiation, strong light, low/high temperature, drought, etc. [52]. These conditions can cause the creation of free radicals due to the stress they cause to the plants, and one of the functions of flavonoids in plants is the reduction of the effect caused by the presence of reactive oxygen species (ROS) [2]. It is possible that luteolin can protect plants from abiotic and biotic stresses. Also, it was proposed that flavonoids, where luteolin belongs, can function as signal molecules, phytoalexins, allopathic compounds, detoxifying agents, antimicrobial defensive compounds and UV filters [53]. They also protect plants from drought, heat and freezing [54,55].

Fertilization is a factor that can affect the concentration of flavonoids. For example, luteolin-7-*O*-glucoside was found to be higher at the first growth stage and also at the 150 kg N ha^−1^ treatment (Table 2) [52]. In addition, crop management, such as organic and conventional, can affect luteolin content. Particularly, luteolin concentration was higher by 51% in the organic system compared with the conventional one [56]. In the same study, luteolin content was increased by 39% with the application of 20 kg N ha^−1^ (1.741 mg 100 g^−1^ dry weight) compared to 0 kg N ha^−1^ (1.383 mg 100 g^−1^ dry weight). Regarding the effect of N fertilization on the organic management system, it was reported that there were no significant changes [56]. Moreover, in another work, the effect of genotype, the ripening stage and their interactions in different growing systems (organic and conventional) were studied in different species of *Capsicum*. It was observed that the ripening stage significantly affected the concentration of luteolin; specifically, luteolin content appeared higher in ripening fruits, as well as in the organic system [57]. 

In a field experiment carried out in Pisa, Italy, different amounts of nitrogen fertilizer were applied to *Stevia rebaudiana*: N0 (without N fertilization), N50 (50 kg N ha^−1^), N150 (150 kg N ha^−1^), N300 (300 kg N ha^−1^) and N_org_ (150 kg N ha^−1^, as organic nitrogen from N_utex_ N7 based on wool wastes, poultry manure, blood, and pomace with 7% organic N and 35% organic carbon). In addition, leaf samplings were accomplished at three vegetative stages (H1, H2, and H3), where H1 and H2 were during the vegetative growth and H3 at the flowering stage. The experiment showed that luteolin-7-*O*-glucoside was significantly affected by the amount of nitrogen fertilization (N), harvest time (H), and NxH interaction. Luteolin-7-O-glucoside was higher in the H1 compared to the other harvests with 150 kg N ha^−1^ (Table 2). In H2, a decrease in Luteolin-7-*O*-glucoside was recorded compared to H1, while in H3, the amount of this compound increased in each N treatment. Values similar in H1 were recorded in the N0 and Norg treatments [52].

Abiotic stress can affect the concentration of luteolin as it was found that water stress was among the main factors that increase the content of secondary metabolites, especially polyphenols/flavonoids [58,59,60]. Water stress increased the concentration of luteolin in *Chrysanthemum* plants [60]. In addition, another study conducted with *Lactuca sativa* plants found that drought stress and UV-B affected the flavonoid content, such as quercetin, luteolin and anthocyanin, as there was an increase in luteolin content under water stress [61]. 

One of the most common abiotic stresses is salinity, as it affects many agricultural areas worldwide (Vafadar et al., 2020). Also, salinity stress affects the secondary metabolism, and it was found that the concentration of phenolic and flavonoid compounds increased [62,63,64]. In addition, it was found that the concentration of luteolin was increased by up to 75 mM NaCl in *Dracocephalum kotschyi* [65]. 

Agati et al. [66] reported that salinity and UV radiation significantly enhanced the biosynthesis of luteolin 7-*O*-glycosides. Other researchers studied the effect of salt stress using *Solanum nigrum* seedlings, and the results of the experiment showed increased flavonoid accumulation and decreased root and leaf dry biomass in the treatment with the highest salt concentration [67]. Similar results were reached by other researchers [68,69].

The growth stage of the plants can affect the flavonoid content and especially the content of luteolin, as it was found by several studies in *Origanum majorana*. Specifically, the highest content is found at the bud stage, as well as in the early vegetative stages and at full bloom [70,71,72].

## 4. Methods of Luteolin Extraction from Different Plant Species

There are various extraction techniques that are reported in the literature and that have been used by many research groups in order to efficiently acquire luteolin and/or luteolin-enriched extracts. Both conventional and modern methods have been applied for extracting luteolin from different plant sources. From maceration and Soxhlet extraction to ultrasound-assisted and microwave-assisted extraction, each technique offers several advantages while exhibiting certain limitations. The choice of the extraction method is highly dependent on the quantity and characteristics of the plant material, as well as the properties of the bioactive compound(s) to be extracted.

### 4.1. Conventional Extraction Methods

Blekas et al. performed the extraction of luteolin from freeze-dried table olive fruits while using the maceration technique and 80% (*v*/*v*) ethanol (containing 0.5% *v*/*v* sodium metabisulfite) as a solvent. The extraction process lasted 20 min and was repeated three times. Luteolin was found to be present at concentrations ranging from 1 to 74 mg/kg of fresh weight in the different table olive samples [36]. Mitsopoulos and co-workers collected samples of leaves and drupes of several olive varieties (e.g., Koroneiki, Kalamata, Konservolia, Chalkidiki) and extracted them using methanol by means of mechanical homogenization. The chromatographic analysis of the extracts showed the presence of 4-*O*-glucoside and 7-*O*-glucoside of luteolin at concentrations of 0.07–1.60 mg/g and 0.11–2.03 mg/g of fresh weight, respectively [40]. In another study, different solvents of increasing polarity (petroleum ether, dichloromethane, methanol and methanol/water 60:40 *v*/*v*) were used to extract samples of olive tree leaves of the Greek cultivars Koroneiki, Megaritiki, and Kalamon that were collected from Thessaloniki, Greece. Luteolin 7-*O*-glucoside was among the main constituents that were detected in the leaf extracts [38]. Samples of virgin olive oil from Southern Greece were extracted with a mixture of ethanol/water (80:20 *v*/*v*), resulting in extracts containing luteolin (0.31–1.29 mmol/100 g of virgin oil) [37]. Kotsiou and Tasioula-Margari [39] performed a liquid–liquid extraction by mixing samples of extra virgin olive oil that were collected from areas of Western Greece with methanol. The analysis of the extracts demonstrated the presence of luteolin at a concentration range of 0.11–1.69 mg/kg olive oil. 

Samples of *C. cardunculus* leaves were extracted with methanol/water (80:20 *v*/*v*) under mild stirring at room temperature. A series of luteolin derivatives were identified in the extract, namely luteolin 7-*O*-rutinoside, luteolin 7-*O*-glucoside and luteolin 7-*O*-malonylglycoside [28]. Similarly, *Cynara humilis* and *C. cornigera* leaves were extracted with ether at room temperature, yielding luteolin and a series of derivatives, such as luteolin 7-*O*-β-d-glucoside and luteolin 7-*O*-β-d-rutinoside [29].

Miceli et al. performed the extraction of flowers and leaves of *Nepeta sibthorpii* by using 95% (*v*/*v*) methanol. The resulting data showed that the methanolic extract contained luteolin at a concentration of 0.387 mg/g of dry weight [33].

Luteolin (0.5 mg/g of dry weight) was also detected in the flowers of *Asphodelus ramosus* that were collected from the Campus of the University of Athens, Greece. In specific, the dried flowers were macerated under agitation with a mixture of methanol/water (5:1 *v*/*v*) [25].

Barda et al. collected the aerial parts of *Crepis incana* from Central Greece and extracted them by using two types of solvents successively. A mixture of cyclohexane/diethyl ether/water (1:1:1 *v*/*v*/*v*) and a mixture of methanol/water (5:1 *v*/*v*) were used as solvents. Luteolin (0.13 mg/g of extract) and luteolin 7-*O*-β-d-glucopyranoside (0.22 mg/g of extract) were detected in the extract [26].

Samples of *Laurus nobilis* leaves that were collected from Northern Greece were macerated with methanol under agitation, leading to a methanolic extract containing luteolin at a quantity of 393.4 μg/g of dry weight [31].

Samples of *Mentha spicata* and *Origanum vulgare* leaves were collected from Southern Greece and ground before extracting them via maceration with a mixture of ethanol/water (10:90 *v*/*v*) at room temperature for 14 days. Luteolin was detected in both plant species, with its content being 19.89 μg/mL of extract and 25.97 μg/mL of extract for *M. spicata* and *O. vulgare*, respectively [32].

Different luteolin derivatives (luteolin 3′-*O*-β-d-glucoside, luteolin 3′,4′-di-*O*-β-d-glucopyranoside) along with the parent compound were identified in a methanolic extract of *Paeonia clusii*. The extract was acquired by macerating *P. clusii* seeds with methanol at room temperature for 24 h. The content of luteolin and its two derivatives was between 0.33 and 0.69 mg/g of dry weight [44].

Chinou and Harvala proceeded to the extraction of *C. humilis* and *C. cornigera* leaves with a Soxhlet apparatus by successively using 95% (*v*/*v*) and 50% (*v*/*v*) ethanol. The analysis showed that luteolin 7-*O*-β-gentiobioside was detected in both species [29]. 

Luteolin was also identified in the extracts of *O. vulgare* and *S. fruticosa*. Both plants were extracted via Soxhlet extraction, while ethanol and acetone were selected as extracting solvents. However, the quantity of luteolin was below the quantitative detection limit based on HPLC/DAD analysis [43]. 

In another study, fresh and dried *Pistacia lentiscus* leaves were extracted for 3–4 h with a Soxhlet apparatus and ethanol or water as solvents. Soxhlet extraction of fresh leaves with water exhibited the highest yield (31.99%) compared to the dried leaves, as well as to the ethanol extract [47]. 

Choulitoudi and co-workers employed a Soxhlet apparatus for performing the extraction of *Satureja thymbra* leaves. Ethyl acetate and ethanol were used as solvents during the successive extractions. The obtained extracts contained different luteolin derivatives, such as luteolin 7,4′-di-*O*-glucuronide (15.6 g/kg of dry extract), 6-OH luteolin 7,3′-dimethyl ether (11.9–13.5 g/kg of dry extract) and 6-OH luteolin 7,3′,4′-trimethyl ether (11.9–13.3 g/kg of dry extract) [50].

Various Greek aromatic plants were extracted with the heat reflux method. Specifically, dried samples of *Cuminum cyminum*, *Nepeta cataria*, *Petroselinum sativum*, *Rosmarinus officinalis*, *Salvia officinalis*, *Spartium junceum*, *Teucrium chamaedrys*, *Teucrium polium* and *Thymus vulgaris* were mixed with methanol/water (62.5:37:5 *v*/*v*), containing 1 g/L butylated hydroxytoluene, and then refluxed in a water bath at 90 °C for 2 h. Luteolin was detected in all plant extracts, with T. vulgaris demonstrating the highest concentration (36 mg/100 g of dry weight) [27,46].

### 4.2. Modern Extraction Methods

Cvetkovikj et al. [49] performed an ultrasound-assisted extraction of Greek *Salvia fruticosa* and *Salvia pomifera* species. Particularly, milled leaves were mixed with 70% (*v*/*v*) methanol and sonicated at 40 °C for 20 min. The chromatographic analyses indicated that the methanolic extract of *S. fruticosa* was abundant in luteolin 7-*O*-rutinoside (4.84 mg/g of dry weight) and luteolin diglucuronide (6.18 mg/g of dry weight), while this was not the case for *S. pomifera*, where the respective values for the luteolin derivatives were <1.00 mg/g of dry weight. *S. fruticosa* was also extracted through a technique combining ultrasonication pretreatment and stirred-tank extraction. Aerial parts of *S. fruticosa* were acquired from a retail store in Southern Greece, mixed with glycerol/water (60:40 *v*/*v*) and sonicated in an ultrasonic bath at 50 °C for 40 min. Next, the mixture was placed in an oil bath at 50 °C for 150 min, under stirring. For comparison reasons, the stirred-tank extraction was also performed at 80 °C and with aqueous solutions containing methyl β-cyclodextrin as a booster for extracting polyphenols. The results showed that luteolin 7-*O*-glucuronide was among the most abundant polyphenols with quantities of 5.51 mg/g of dry weight (60% *v*/*v* glycerol) and 6.96 mg/g of dry weight (methyl β-cyclodextrin). Other luteolin derivatives (6-hydroxy luteolin 7-*O*-glucoside, luteolin 7-*O*-rutinoside, 6-methoxyluteolin 7-*O*-glucoside, 6-methoxyluteolin derivative and 6-methoxyluteolin derivative) were tentatively identified as well, yet their concentrations were significantly low [48].

Another research group extracted freeze-dried olives (Kalamon cultivar) three times by using 80% (*v*/*v*) acetone and an ultrasonic ice bath for 15 min. The extract contained luteolin (0.04 mg/g of dry weight) and its 7-*O*-glucoside (0.21 mg/g of dry weight) [42]. Similarly, Salis et al. [41] used fresh Kalamon olives for extracting luteolin. The extraction process involved the mechanical homogenization of olives with hexane, followed by sonication in a supersonic bath with 80% (*v*/*v*) methanol for 30 min. The sonication step was repeated four times in total. The HPLC analysis of the extracts revealed that luteolin content ranged between 92.4 and 118.16 μg/g of fresh weight. Luteolin was also extracted via sonication from *Juglans regia* septa and *Petroselinum crispum* leaves. Powdered *J. regia* septa were mixed with methanol/water (60:40 *v*/*v*) containing 0.05% (*v*/*v*) trifluoroacetic acid and sonicated in an ultrasonic bath for 10 min at 25 °C. On the other hand, *P. crispum* leaves were freeze-dried, powdered and mixed with 80% (*v*/*v*) methanol for 3 h at room temperature prior to sonication (30 min). Once sonication was completed, the extraction continued via maceration overnight at 4 °C in the dark. The content of luteolin in *J. regia* septa was 2.4–3.4 μg/g of dry weight, while in *P. crispum* leaves 0.13–0.15 mg/100 g of dry weight [30,45]. In addition to the above, Boutsika et al. detected luteolin 7-*O*-glucoside (0.27–0.45 mg/100 g of dry weight) and 3′-methyl-luteolin (0.22–0.32 mg/100 g of dry weight) in *P. crispum* extract [45].

## 5. Pharmacological Activities and Applications of Luteolin

The different pharmacological activities of luteolin have been well described in the literature. Luteolin exhibits a series of biological activities, such as cytotoxic, anti-inflammatory, antioxidant and antibacterial ones. Some of the possible mechanisms through which luteolin exerts its therapeutic effects are the following: preventing DNA alterations in oncogene and tumor-suppressor genes, decreasing vascular endothelial growth factor (VEGF), inhibiting the elevation of reactive oxygen species (ROS), and downregulating inflammatory cytokines (TNF-α) and transcription factors (Nf-κB, STAT3) [21]. Moreover, luteolin, as an anti-inflammatory agent, acts by inhibiting the activation of mast cells and T cells in cases of neuroinflammation and allergic inflammation [73,74,75,76].

In a recent study, luteolin was also found to inhibit in vitro and in vivo (C57BL/6J mice) the activation of NOD-, LRR- and pyrin domain-containing protein 3(NLRP3) inflammasome, therefore supporting luteolin’s role as an anti-inflammatory agent [77]. Moreover, Wang and co-workers demonstrated that luteolin could modify the M1/M2 polarization of macrophages and exert its anti-inflammatory role by decreasing p-STAT3 and increasing p-STAT6 [78]. Alternatively, luteolin reduced the inflammation in an acute gouty arthritis rat model by negatively regulating the TLR/MyD88/NF-κB pathway [79]. Researchers also reported that luteolin protected retinal pigment epithelium cells from increasing interleukin levels –and thereby, inflammation– through inhibition of MAPK and NF-kΒ [80]. Last but not least, luteolin protects ulcerative colitis rats by reducing inflammation and enhancing the composition and diversity of gut microbiota [81]. In a different animal model (TG-AD mice), luteolin was found to exert a protective effect against Alzheimer’s disease through the inhibition of endoplasmic reticulum stress-associated neuroinflammation [82]. A common luteolin derivative, luteolin 7-*O*-glucoside, showed promising in vitro anti-inflammatory properties by suppressing ROS and STAT3 activation in HUVEC cells [55].

The antioxidant and antibacterial activities of luteolin have been recently reported by different groups, demonstrating the ability of this molecule to reduce ochratoxin-stimulated oxidative stress in vitro through Nrf2 and HIF-1α pathways and suppressing the growth of *Trueperella pyogenes* by disrupting cell wall and cell membrane integrity, hindering the synthesis of nucleic acids, altering metabolism and modulating protein expression [83,84].

**Figure 3:**
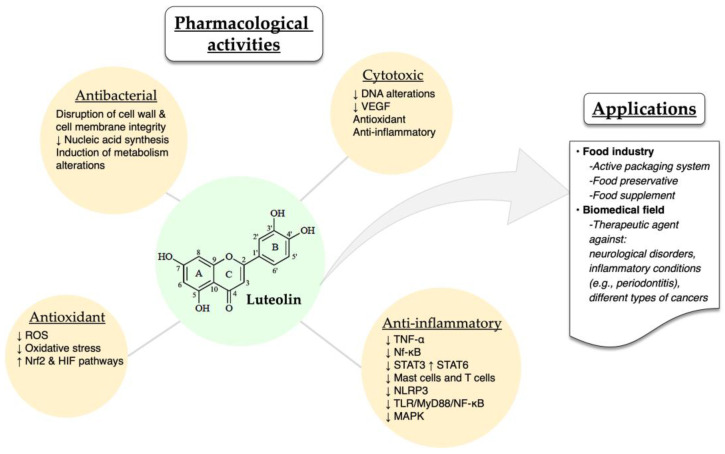
Overview of the pharmacological activities and applications of luteolin. The up and down arrows indicate increases and decreases, respectively.

Due to its biological potential, luteolin has been employed as a bioactive molecule in various applications within the food industry and the biomedical field. For instance, luteolin has been incorporated into the active packaging system of different foods as well as an additive [85]. Bi et al. [86] demonstrated that luteolin nanoemulsions displayed potent antioxidant properties, therefore protecting fat-containing foods, such as beef, poultry and fish (Figure 3). Additionally, luteolin acted as a food preservative when added to minced meat by hindering the development of *Listeria monocytogenes* [87]. Also, a commercially available dietary supplement that contains luteolin, rutin and quercetin was shown to exert an antioxidant effect. Other therapeutic applications of luteolin include the employment of different delivery platforms (micelles, liposomes, nanoemulsions, amorphous solid dispersions) with the aim to increase the active molecule’s bioavailability and its overall therapeutic efficacy [88,89,90,91]. Luteolin and luteolin-containing drug delivery systems have been studied for the treatment of numerous diseases, such as neurological disorders [92], rheumatoid arthritis [93] and different types of cancers [94,95,96] (Figure 3).

## 6. The Effect of Luteolin in the Prevention and Treatment of Periodontal Disease

Among the various available options for managing periodontitis, dental care products containing herbal compounds have been in the spotlight owing to the beneficial pharmacological properties of the bioactive ingredients [97]. In this context, the anti-inflammatory activity of luteolin has been harnessed in order to combat periodontal disease and promote the restoration of damaged bone tissue. Gutiérrez-Venegas and co-workers demonstrated that luteolin could hinder the inflammation in lipopolysaccharide-stimulated human gingival fibroblasts by downregulating a series of mitogen-activated protein kinase family members [98]. In a different study, Wistar rats were used as a model for assessing the use of luteolin in periodontitis prevention. The results suggested that luteolin succeeded in reducing the inflammation and potentially induced osteoblast differentiation [99]. Moreover, it was found that luteolin could facilitate osteogenic differentiation in human periodontal ligament cells through the activation of the Wnt/β–catenin signaling pathway [100]. Casili et al. confirmed that luteolin could alleviate periodontitis symptoms in Sprague-Dawley rats by reducing inflammation through the reduction of TNF-α and IL-6 expression [23]. In relation to the anti-inflammatory activity of luteolin, which is relevant in periodontitis treatment, both in vitro and in vivo studies have shown that luteolin inhibits many pro-inflammatory cytokines (like TNF-α), as well as modulate NF-κB pathway [101]. 

Luteolin’s antimicrobial properties have been well documented. In some studies, it was attempted to isolate the bioactive compounds of several flavonoids while examining their antimicrobial activities against oral bacteria related to the establishment of periodontitis. Luteolin appeared to exhibit great antimicrobial activity against the oral microbes tested (including *Porphyromonas gingivalis*) due to the presence of hydroxyl group at the third position [102]. More specifically, it inhibited bacterial growth, leading to a reduction in the total count of bacteria. Taken together, our collected data suggest that luteolin has the potential, as a useful adjunct agent, to regulate both prevention and treatment of periodontal diseases.

## 7. Conclusions

Flavonoids are a class of naturally occurring compounds that are present in a plethora of aromatic plants. More specifically, they are a group of low molecular weight polyphenols being produced as secondary metabolites in plants. These compounds have attracted a great amount of interest because of their important biological activities and their beneficial effect on human health. Luteolin is a bioactive flavonoid that also belongs to the subgroup of flavones and can be found in several plants, such as the genera *Cynara*, *Origanum* and *Salvia*, among others. Greek flora is abundant with a vast number of aromatic plants that may present a good source of luteolin and/or its derivatives. This review provided a comprehensive overview of the different luteolin-containing Greek plant species, the employed conventional and modern extraction techniques for obtaining luteolin and/or its derivatives, and the analytical methods for detecting these bioactive compounds. Additionally, the up-to-date literature data concerning crop management (e.g., fertilization, irrigation) in relation to the production of luteolin (or its derivatives) in plants has been discussed. Overall, the findings of the current review suggest that Greek plants could be used for isolating adequate quantities of luteolin and further utilizing it for developing oral formulations against periodontal disease.

## Figures and Tables

**Table 1:** Content of luteolin and/or its derivative(s) in different plant species of the Greek flora.

Plant Species	Family	Active Compound(s)	Concentration of Active Compound(s)	Reference
*Apium graveolens*	Apiaceae	luteolin luteolin-7-*O*-glucoside	2.31 mg/100 g FW 654 mg/100 g DW	[24]
*Asphodelus ramosus*	Asphodelaceae	luteolin	0.5 mg/g DW	[25]
*Crepis incana*	Asteraceae	luteolin, luteolin 7-*O*-β-d-glucopyranoside	0.13 mg/g EXTR 0.22 mg/g EXTR	[26]
*Cuminum cyminum*	Apiaceae	luteolin	3.1 mg/100 g DW	[27]
*Cynara cardunculus*	Asteraceae	luteolin 7-*O*-rutinoside, luteolin 7-*O*-glucoside, luteolin 7-*O*-malonylglycoside	1.31–7.8 mg/g EXTR 1.52–8.5 mg/g EXTR 0.52–11.8 mg/g EXTR	[28]
*Cynara humilis*, *Cynara cornigera*	Asteraceae	luteolin, luteolin 7-*O*-β-d-glucoside, luteolin 7-*O*-β-d-rutinoside, luteolin 7-*O*-β-gentiobioside	NQ NQ NQ NQ	[29]
*Juglans regia*	Juglandaceae	luteolin	2.4–3.4 μg/g DW	[30]
*Laurus nobilis*	Lauraceae	luteolin	393.4 μg/g DW	[31]
*Mentha spicata*	Lamiaceae	luteolin	19.89 μg/mL EXTR	[32]
*Nepeta cataria*	Lamiaceae	luteolin	3.6 mg/100 g DW	[27]
*Nepeta sibthorpii*	Lamiaceae	luteolin 7-*O*-glucoside	0.387 mg/g DW	[33]
*Ocimum basilicum*	Lamiaceae	luteolin 7-*O*-glucoside	127 mg/100 g DW	[34]
*Olea europaea*	Oleaceae	luteolin	NQ	[35]
*Olea europaea*	Oleaceae	luteolin	1–74 mg/kg FW	[36]
*Olea europaea*	Oleaceae	luteolin	0.31–1.29 μmol/100 g virgin olive oil	[37]
*Olea europaea*	Oleaceae	luteolin 7-*O*-glucoside	NQ	[38]
*Olea europaea*	Oleaceae	luteolin	0.11–1.69 mg/kg virgin olive oil	[39]
*Olea europaea*	Oleaceae	luteolin 4-*O*-glucoside, luteolin 7-*O*-glucoside	0.07–1.60 mg/g FW 0.11–2.03 mg/g FW	[40]
*Olea europaea*	Oleaceae	luteolin	92.4–118.16 μg/g FW	[41]
*Olea europaea*	Oleaceae	luteolin, luteolin 7-*O*-glucoside	0.04 mg/g DW 0.21 mg/g DW	[42]
*Origanum majorana*	Lamiaceae	luteolin 7-*O*-glucoside	461 mg/g DW	[34]
*Origanum vulgare*	Lamiaceae	luteolin	<LOQ	[43]
*Origanum vulgare*	Lamiaceae	luteolin	25.97 μg/mL EXTR	[32]
*Paeonia clusii*	Paeoniaceae	luteolin, luteolin 3′-*O*-β-d-glucoside, luteolin 3′,4′-di-*O*-β-d-glucopyranoside	0.69 mg/g DW 0.33 mg/g DW 0.35 mg/g DW	[44]
*Petroselinum crispum*	Apiaceae	luteolin, luteolin 7-*O*-glucoside, 3′-methyl-luteolin	0.13–0.15 mg/100 g DW 0.27–0.45 mg/100 g DW 0.22–0.32 mg/100 g DW	[45]
*Petroselinum sativum*	Apiaceae	luteolin	2.1 mg/100 g DW	[46]
*Pistacia lentiscus*	Anacardiaceae	luteolin	NQ	[47]
*Rosmarinus officinalis*	Lamiaceae	luteolin	1.6 mg/100 g DW	[46]
*Salvia fruticosa*	Lamiaceae	luteolin	<LOQ	[43]
*Salvia fruticosa*	Lamiaceae	6-hydroxy luteolin 7-*O*-glucoside, luteolin 7-*O*-glucuronide, luteolin 7-*O*-rutinoside, 6-methoxyluteolin 7-*O*-glucoside, 6-methoxyluteolin derivative, 6-methoxyluteolin derivative	<LOQ <LOQ 5.51–6.96 mg/g DW <LOQ <LOQ <LOQ	[48]
*Salvia fruticosa*, *Salvia pomifera*	Lamiaceae	luteolin 7-*O*-rutinoside, luteolin diglucuronide	4.84 mg/g DW 6.18 mg/g DW	[49]
*Salvia officinalis*	Lamiaceae	luteolin	0.2 mg/100 g DW	[46]
*Satureja thymbra*	Lamiaceae	luteolin 7,4′-di-*O*-glucuronide, 6-OH luteolin 7,3′-dimethyl ether, 6-OH luteolin 7,3′,4′-trimethyl ether	15.6 g/kg DE 11.9–13.5 g/kg DE 11.9–13.3 g/kg DE	[50]
*Spartium junceum*	Fabaceae	luteolin	2.5 mg/100 g DW	[27]
*Teucrium chamaedrys*	Lamiaceae	luteolin	1.2 mg/100 g DW	[46]
*Teucrium polium*	Lamiaceae	luteolin	0.48 mg/100 g DW	[51]
*Thymus vulgaris*	Lamiaceae	luteolin	36 mg/100 g DW	[46]

DE, dry extract; DW, dry weight; EXTR, extract; FW, fresh weight; LOQ, limit of quantification; NQ, no quantification.

**Table 2:** Effect of Nitrogen Fertilization and Harvest Time on Luteolin-7-*O*-glucoside in Leaves of *Stevia rebaudiana*.

Luteolin-7-*O*-glucoside (mg g^−1^ DW)					
	N0	N50	N150	N300	Norg
H1	45.67 *	45.28	76.61	36.46	35.20
H2	16.04	17.85	19.93	12.15	19.16
H3	47.30	22.18	16.83	26.08	30.00

* Results are the means of five replicates that were each analyzed in triplicate. Where N0 is the treatment without N fertilization; N50 is a treatment with 50 kg N ha^−1^; N150 is a treatment with 150 kg N ha^−1^; N300 is a treatment with 300 kg N ha^−1^; Norg is a treatment with 150 kg N ha^−1^ as organic nitrogen. H1 is the first harvest time on 9 July, H2 is the second harvest time on July 21, and H3 is the third harvest time on 10 September. Adapted from Tavarini et al. [52].

## Data Availability

Not applicable.
